# The dynamic trajectory of symptom networks in patients with acute pancreatitis during hospitalization

**DOI:** 10.3389/fmed.2025.1723941

**Published:** 2026-01-12

**Authors:** Xue Yang, Hairong Lin, Li Zhang, Jin Chen, Hong Li, Decai Wang, Guirong Li

**Affiliations:** 1 Department of Gastroenterology, Mianyang Central Hospital, School of Medicine, University of Electronic Science and Technology of China, Mianyang, Sichuan, China; 2 Department of Hepatobiliary Surgery, Mianyang Central Hospital, School of Medicine, University of Electronic Science and Technology of China, Mianyang, Sichuan, China; 3 Nursing Department, Mianyang Central Hospital, School of Medicine, University of Electronic Science and Technology of China, Mianyang, Sichuan, China

**Keywords:** acute pancreatitis, network analysis, nursing, nursing management, symptom cluster

## Abstract

**Background:**

To identify the composition and dynamic changes of symptom clusters in hospitalized acute pancreatitis (AP) patients, and to explore core and bridge symptoms via symptom network analysis, thereby providing a basis for precise symptom management.

**Methods:**

A total of 194 AP patients admitted to a tertiary hospital in Mianyang, Sichuan from September 2024 to September 2025 were included. General information and the MD Anderson Symptom Inventory-Gastrointestinal Module were used for daily assessment. Exploratory factor analysis was applied to identify symptom clusters, and symptom networks were constructed using R software to analyze centrality indices.

**Results:**

Three symptom clusters were identified: gastrointestinal symptom cluster, sleep-fatigue symptom cluster, and oropharyngeal-psychological distress symptom cluster. The acute pancreatitis symptom network exhibits a dynamic shift during hospitalization, evolving from an initial predominance of gastrointestinal symptoms to a later profile characterized by oropharyngeal-psychological discomfort and sleep-fatigue clusters. At T0, nausea had the highest strength centrality (rs = 1.698), while fatigue was the strongest bridge symptom (rb = 0.95). At T1, abdominal pain (rs = 1.302) and pain (rb = 0.913) were the most central and bridging symptoms, respectively. At T2, dry mouth (rs = 1.018) and bloating (rb = 0.683) exhibited the highest values. By T3, fatigue (rs = 1.621) and pain (rb = 1.59) again showed the highest centrality and bridge strength. Fatigue and abdominal pain are persistent core symptoms, with pain also serving as a crucial bridge symptom.

**Conclusion:**

Symptom experiences in AP patients change dynamically during hospitalization. Targeting core and bridge symptoms can enhance precision and efficiency in symptom management, reducing the overall symptom burden.

## 1 Introduction

Acute pancreatitis (AP) is a frequently encountered clinical emergency affecting the digestive system (1). The global incidence of acute pancreatitis is 34 per 100,000 person-years and is increasing annually (2). Acute pancreatitis is characterized by its sudden onset, rapid progression, diverse symptoms, and significant disease burden. Patients frequently experience adverse symptoms such as abdominal pain, bloating, and fatigue (3), which can significantly impair their quality of life and are indicative of greater disease severity. For example, studies have shown that pain induces physiological distress, triggering severe psychological stress and promoting the secondary release of neurogenic mediators, which in turn exacerbates pancreatic tissue injury (4). The sensation of symptoms such as dry mouth can also induce a stress response, which increases the body’s oxygen consumption and the metabolic burden on organs, thereby ultimately influencing the disease outcome (5). Furthermore, the debilitating symptom experience often evokes negative emotional states, including anxiety, fear, and depression, which significantly diminish the quality of life and impede recovery (6). Evidence indicates a high prevalence of these psychological comorbidities, with anxiety affecting 29% and depression 35.7% of AP patients (7). Notably, a case-control study revealed that the distressing symptoms and subsequent mood disorders associated with AP can elevate the risk of suicide (8). Consequently, the precise management of symptoms during hospitalization for Acute Pancreatitis is crucial to reduce the overall symptom burden. However, the current understanding of patient symptoms remains insufficient. Management strategies often focus on isolated symptoms, such as pain or anxiety, while neglecting the interconnections between symptoms and the fact that they frequently present as symptom clusters. A symptom cluster is defined as two or more concurrent symptoms that are highly predictable and closely interrelated (9). The clinical significance of identifying symptom clusters lies in their ability to elucidate the relationships between symptoms, thereby enabling a more comprehensive and efficient reduction of the patient’s symptom burden (10, 11). Luo et al. (12) demonstrated that symptom cluster management in patients with lung cancer effectively reduced symptom burden and improved quality of life. Qiu et al. (13) showed that implementing symptom cluster management for heart failure patients is beneficial in alleviating the disease’s burden on mental cognition and fatigue. In addition, network analysis, as an emerging method, also holds great promise in the symptom domain, because it can visualize the associations between symptoms and, by calculating centrality strength, identify important symptoms among them, thereby indicating targets for symptom intervention (14). Zhou et al. (15) have enhanced the efficiency and precision of symptom management by identifying core symptoms and pinpointing intervention targets in stroke patients. Research has demonstrated that applying network analysis to symptom management in lung cancer patients undergoing immunotherapy identified dizziness as the most central symptom, with fatigue serving as a critical bridging element. These findings directly inform clear intervention priorities, providing a scientific basis for developing highly efficient and precise personalized symptom management protocols (16). Thus, the present study is designed to employ network analysis to investigate the relationships among symptoms in AP inpatients, to identify core and bridge symptoms, and to furnish an evidence base for precise clinical symptom intervention.

## 2 Design and methods

### 2.1 Study subjects

This study adopted a convenience sampling method, taking patients with acute pancreatitis hospitalized in a Grade A tertiary hospital in Mianyang City from September 2024 to September 2025 as study subjects. Inclusion criteria: (1) Aged ≥ 18 years. (2) A patient diagnosed with acute pancreatitis. (3) Course of illness from onset within 24 h. (4) Consenting to participate in this study and signing the informed consent form. Exclusion criteria: (1) Those with other severe diseases, such as cardiac, hepatic, pulmonary, or renal insufficiency, and malignant tumors, etc. (2) Those in a state of pregnancy or lactation. (3) Those with cognitive dysfunction. (4) Those with communication barriers and unable to complete this study.

### 2.2 Sample size calculation

The sample size for this study was estimated based on requirements for network analysis (17–19). For a network with p nodes, the sample size is calculated as P(P–1)/2. In this study, there were 10 nodes with a symptom incidence greater than 20%. According to the formula [10 × (10^−1^)/2], a minimum of 45 participants was required. Allowing for 10% invalid responses, the final calculated sample size was 50. A total of 194 patients were ultimately included in the study, which met the sample size requirement.

### 2.3 Data collection

#### 2.3.1 General information

Patient data were collected using a self-designed general information questionnaire. This included sociodemographic characteristics such as sex, age, height, and weight, as well as disease-related characteristics including smoking history, alcohol consumption history, medical history, classification of pancreatitis type, and severity of pancreatitis—assessed according to the revised Atlanta classification criteria (2012) for acute pancreatitis (AP) (20).

#### 2.3.2 Symptom collection

Symptom assessment was performed using the Chinese version of Part I of the MD Anderson Symptom Inventory–Gastrointestinal Module (MDASI-GI) (21). This instrument measures the severity of common gastrointestinal, psychological, and sleep-related symptoms experienced in the past 24 h. In addition to symptoms frequently seen in acute pancreatitis patients—such as abdominal pain, bloating, nausea, vomiting, dry mouth, and bitter taste—it also covers sleep and psychological disturbances. Each symptom is rated on a numerical scale from 0 to 10, where 0 indicates “not present” and 10 represents “as bad as you can imagine.” The Cronbach’s *α* for Part I of the original scale was 0.84. In this study population, the scale demonstrated good reliability with a Cronbach’s *α* of 0.714.

### 2.4 Data collection methods and ethical approval

This study was conducted in accordance with the Declaration of Helsinki and was approved by the Ethics Committee of a tertiary Grade A hospital in Mianyang, Sichuan Province (Approval No.: S202403142-02). General patient information and symptom assessment scales were collected within 24 h of admission. Symptom data were obtained daily in the afternoon through face-to-face interviews.

### 2.5 Quality control

To ensure data quality, the researcher explained all questionnaire items to patients in plain language. All questionnaires were collected and reviewed immediately; any with missing values were deemed invalid and excluded. Furthermore, a double-data entry system with cross-verification was implemented to guarantee accuracy during the data entry phase.

## 3 Statistical analysis

This study used SPSS 26.0 and R 4.4.3 software for data analysis. Symptoms with an incidence rate >20% were selected for symptom cluster extraction and network analysis.

Symptom cluster extraction: Exploratory factor analysis was performed using SPSS 26.0 to extract symptom clusters from symptoms with an incidence >20% (22). First, the suitability of the symptom data for factor analysis was assessed using the Kaiser–Meyer–Olkin (KMO) test and Bartlett’s test of sphericity. The criteria were set as follows: KMO value >0.7 and Bartlett’s test significance <0.01 to confirm that the data were appropriate for factor analysis. Principal component analysis was used to extract factors, followed by varimax rotation for factor rotation. Factor extraction adhered to the following principles: (1) initial eigenvalues ≥1; (2) symptoms with factor loadings >0.4 were retained, and if a symptom had loadings ≥0.4 on multiple factors, it was assigned to the factor with the highest loading.

Symptom network construction: Based on data from each time point, undirected partial correlation networks were estimated using the EBICglasso method (hyperparameter *γ* = 0.5) (23). Networks were constructed using the ‘estimateNetwork (default = “EBICglasso”)’ function from the bootnet package, with the input correlation matrix computed by the ‘cor_auto()’ function from the qgraph package. Visualization was performed using the ‘qgraph()’ function, with edge display thresholds (minimum = 0.1, cut = 0.2) set to highlight strong associations. The spring layout algorithm was used to generate node positions for the Day 1 network, and this layout was fixed and reused for networks at subsequent time points to ensure consistent node placement across time points for easier comparison. In the visualization, nodes represent symptoms, and edges represent associations—thicker and darker edges indicate stronger partial correlation coefficients (24, 25).

Symptom network evaluation: Centrality measures for each node, including strength, closeness, and betweenness centrality, were obtained using the `centrality()` function from the qgraph package. Bridge strength was calculated using the `bridge()` function from the networktools package to identify key nodes connecting different symptom clusters (factors). The accuracy and stability of the symptom networks were examined using the bootnet package: the reliability of parameter estimates was assessed by computing 95% confidence intervals for edge weights (partial correlation coefficients) via nonparametric bootstrapping (1,000 bootstrap samples); the stability of the network structure was evaluated using the case-dropping bootstrap approach in the bootnet package, with the correlation stability coefficient (CS coefficient) used to measure the stability of centrality indices. A CS coefficient >0.25 was considered acceptable (26). To statistically compare symptom networks across hospitalization days (T0–T3), we performed a Network Comparison Test (NCT) to evaluate differences in both global network structure and connection strength.

## 4 Results

### 4.1 General characteristics of AP patients

A total of 200 patients were enrolled in this study. After excluding six invalid cases (two dropped out and four had missing data), these six cases with missing data were removed using complete case deletion. One hundred ninety-four patients were included for analysis, yielding a valid response rate of 97%. The cohort consisted of 118 men and 76 women. Detailed characteristics are presented (Table 1).

**Table 1:** Baseline characteristics of patients with acute pancreatitis.

Variable	Category	*n* (%)
Sex	Male	118 (60.8%)
	Female	76 (39.2%)
Age (years)	<45	85 (43.8%)
	45–60	78 (40.2%)
	61–75	28 (14.5%)
	>75	3 (1.5%)
Education level	Primary school or below	33 (17%)
	Junior high school	64 (33%)
	High school	34 (17.5%)
	College or above	63 (32.5%)
Etiology	Biliary	32 (16.5%)
	Hyperlipidemic	56 (28.9%)
	Alcoholic	3 (1.5%)
	Idiopathic	103 (53.1%)
Recurrence	Yes	85 (43.8%)
	No	109 (56.2%)
Disease severity	Mild acute pancreatitis	159 (82%)
	Moderately severe acute pancreatitis	31 (16%)
	Severe acute pancreatitis	4 (2%)
Diabetes	Yes	38 (19.6%)
	No	156 (80.4%)
Mild liver disease	Yes	172 (88.7%)
	No	22 (11.3%)
Smoking history	Yes	76 (39.2%)
	No	109 (56.2%)
	Former smoker	9 (4.6%)
Drinking history	Yes	78 (40.2%)
	No	104 (53.6%)
	Former drinker	12 (6.2%)

### 4.2 Occurrence of symptoms and extraction of symptom clusters in AP patients

**Figure 1:**
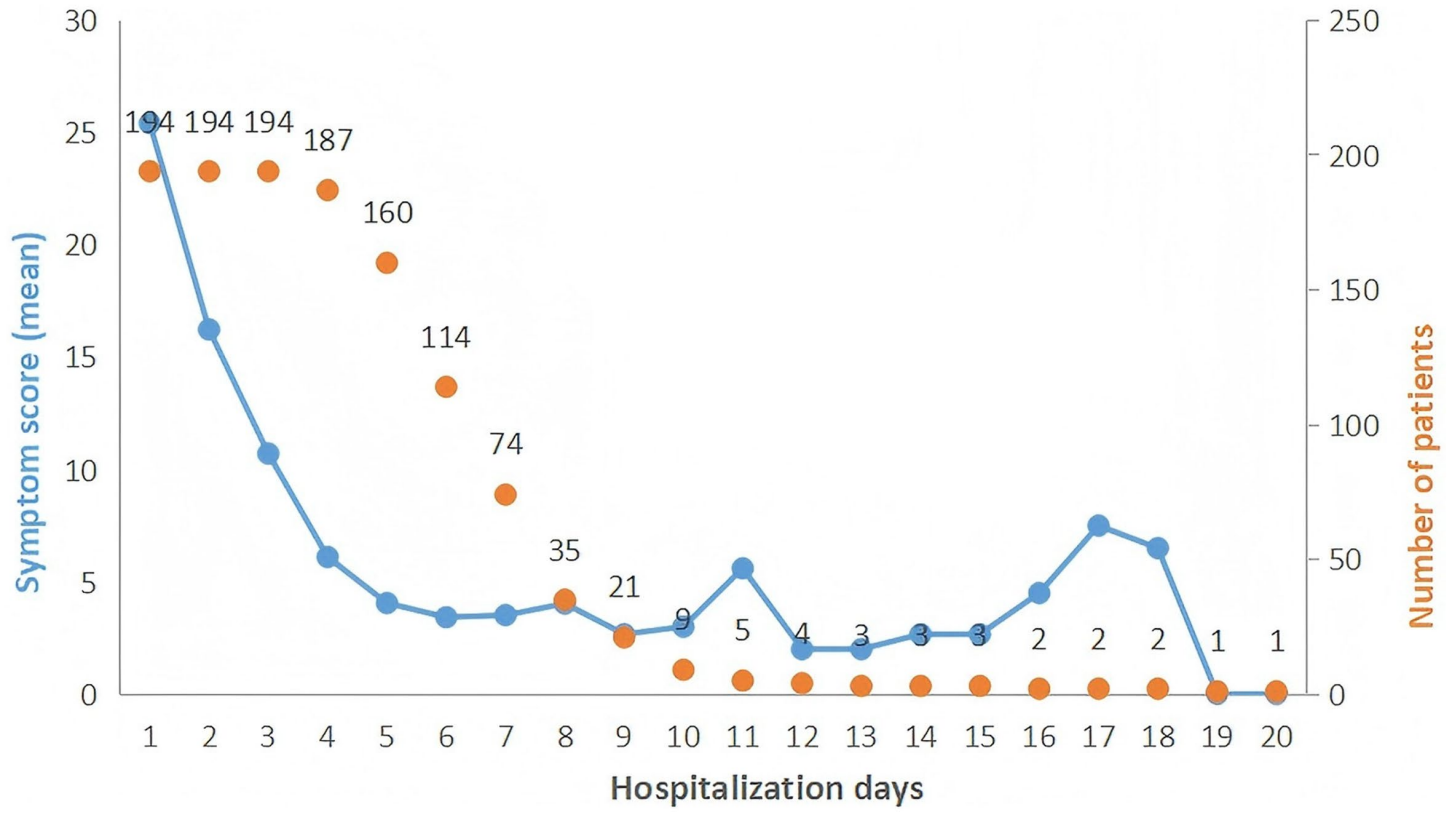
Trends in symptom burden among hospitalized patients with acute pancreatitis.

As shown in the trends in Symptom Burden among Hospitalized Patients with Acute Pancreatitis (Figure 1), symptom scores decreased significantly and stabilized by the fourth hospital day. Therefore, the first 4 days of hospitalization were selected as the time points for analyzing symptom clusters, along with their core and bridge symptoms, in patients with pancreatitis. Given the short-term stability of symptom clusters, the clusters identified on the first day were taken as representative. Three symptom clusters were extracted and named as follows: the gastrointestinal symptom cluster, the sleep-fatigue cluster, and the oropharyngeal-psychological distress cluster. Details are provided in Table 2, and the prevalence of individual symptoms is presented in Table 3. The suitability of the data for factor analysis was confirmed by a Kaiser–Meyer–Olkin (KMO) value of 0.702 and a Bartlett’s test of sphericity with *p* < 0.001. The total variance explained by the extracted clusters was 58.025%. The symptom network structure remained largely stable; however, a significant difference in global connection strength was observed between the first and fourth days of hospitalization, with stronger connections on day 4 (Table 4).

**Table 2:** Factor loadings of symptoms in patients with acute pancreatitis.

Symptom cluster	Symptom composition	Factor loading
Gastrointestinal symptom cluster	Pain	0.494
	Abdominal distension	0.696
	Nausea	0.838
	Vomiting	0.824
Sleep-fatigue symptom cluster	Fatigue	0.774
	Sleep disturbance	0.616
	Drowsiness	0.768
Oropharyngeal-psychological distress symptom cluster	Dry mouth	0.506
	Taste change	0.853
	Distress	0.660

**Table 3:** Symptom incidence at different time points during hospitalization in patients with acute pancreatitis.

Symptom	T0 *n* (%)	T1 *n* (%)	T2 *n* (%)	T3 *n* (%)
Pain	178 (91.7%)*	123 (63.4%)*	83 (42.8%)*	50 (29.4%)*
Abdominal distension	85 (43.8%)*	62 (31.9%)*	45 (23.2%)*	37 (19.1%)
Nausea	73 (37.6%)*	20 (10.3%)	12 (6.1%)	6 (3.1%)
Vomiting	53 (27.3%)*	10 (5.1%)	3 (1.5%)	3 (1.5%)
Shortness of breath	3 (1.5%)	2 (1.0%)	9 (4.6%)	4 (2.0%)
Fatigue	73 (37.6%)*	68 (35.1%)*	52 (26.8%)*	42 (21.6%)*
Restless sleep	124 (63.9%)*	87 (44.8%)*	69 (35.6%)*	59 (30.4%)*
Lethargy	82 (42.2%)*	45 (25.3%)*	33 (17.0%)	24 (12.3%)
Dry mouth	163 (84.0%)*	167 (86.1%)*	126 (64.9%)*	80 (41.2%)*
Altered taste	91 (46.9%)*	95 (49.0%)*	73 (37.6%)*	49 (25.3%)*
Constipation	4 (2.0%)	2 (1.0%)	1 (0.5%)	0 (0%)
Diarrhea	4 (2.0%)	7 (3.6%)	22 (11.3%)	14 (7.2%)
Numbness	0 (0%)	0 (0%)	0 (0%)	0 (0%)
Sadness	21 (10.8%)	11 (5.7%)	6 (3.1%)	4 (2.0%)
Distress	134 (69.0%)*	116 (59.8%)*	96 (49.5%)*	83 (42.8%)*
Dysphagia	0 (0%)	0 (0%)	0 (0%)	0 (0%)
Poor appetite	4 (2.0%)	3 (1.5%)	6 (3.1%)	5 (2.6%)
Forgetfulness	3 (1.5%)	1 (0.5%)	0 (0%)	0 (0%)

T0: Day 1 of hospitalization; T1: Day 2 of hospitalization; T2: Day 3 of hospitalization; T3: Day 4 of hospitalization. *Symptom incidence > 20%.

**Table 4:** Comparison of symptom networks in patients with acute pancreatitis during hospitalization.

Comparison group	Test dimension	*p*-value
T0 vs. T1	1. Network invariance	0.679
	2. Global strength invariance	0.831
	3. Edge strength invariance	All edges *p* > 0.05
T1 vs. T2	1. Network invariance	0.689
	2. Global strength invariance	0.280
	3. Edge strength invariance	All edges *p* > 0.05
T2 vs. T3	1. Network invariance	0.874
	2. Global strength invariance	0.471
	3. Edge strength invariance	All edges *p* > 0.05
T0 vs. T3	1. Network invariance	0.063
	2. Global strength invariance	0.004*
	3. Edge strength invariance	All edges *p* > 0.05

T0: Day 1 of hospitalization; T1: Day 2 of hospitalization; T2: Day 3 of hospitalization; T3: Day 4 of hospitalization.*p > 005.

### 4.3 Symptom network analysis in AP patients during hospitalization

#### 4.3.1 Symptom networks at different time points during hospitalization

Symptoms with an incidence rate greater than 20% were included in the network analysis (Table 3). The results revealed the following patterns:

On admission day 1 (T0), nausea had the highest strength centrality (rs = 1.698), while fatigue exhibited the greatest bridge strength (rb = 0.950).

On day 2 (T1), abdominal pain showed the highest strength centrality (rs = 1.302), and pain had the maximum bridge strength (rb = 0.913).

On day 3 (T2), dry mouth demonstrated the highest strength centrality (rs = 1.018), whereas abdominal distension presented the greatest bridge strength (rb = 0.683).

On day 4 (T3), fatigue again showed the highest strength centrality (rs = 1.621), and pain had the largest bridge strength (rb = 1.590).

**Figure 2:**
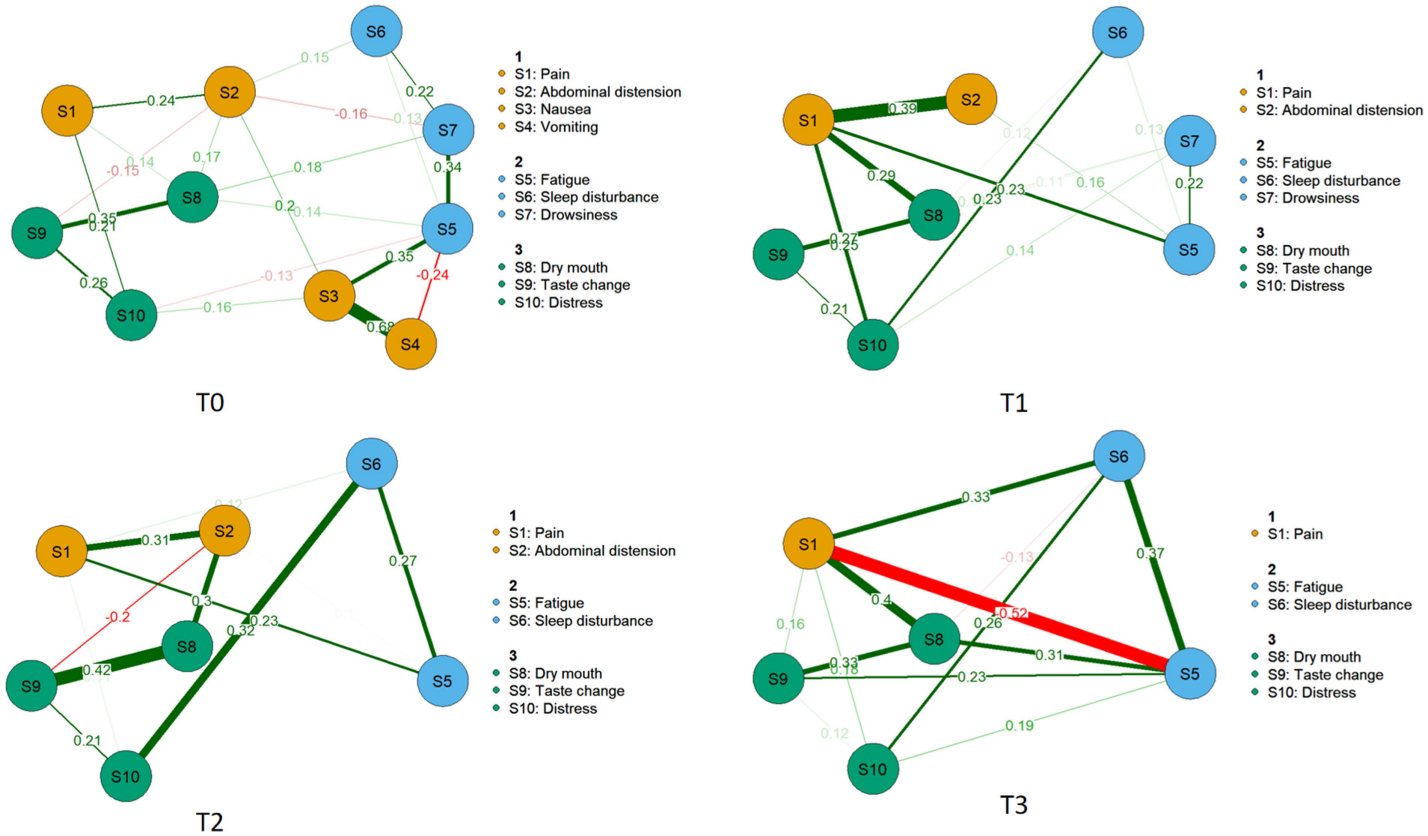
Symptom network in hospitalized patients with acute pancreatitis.

**Figure 3:**
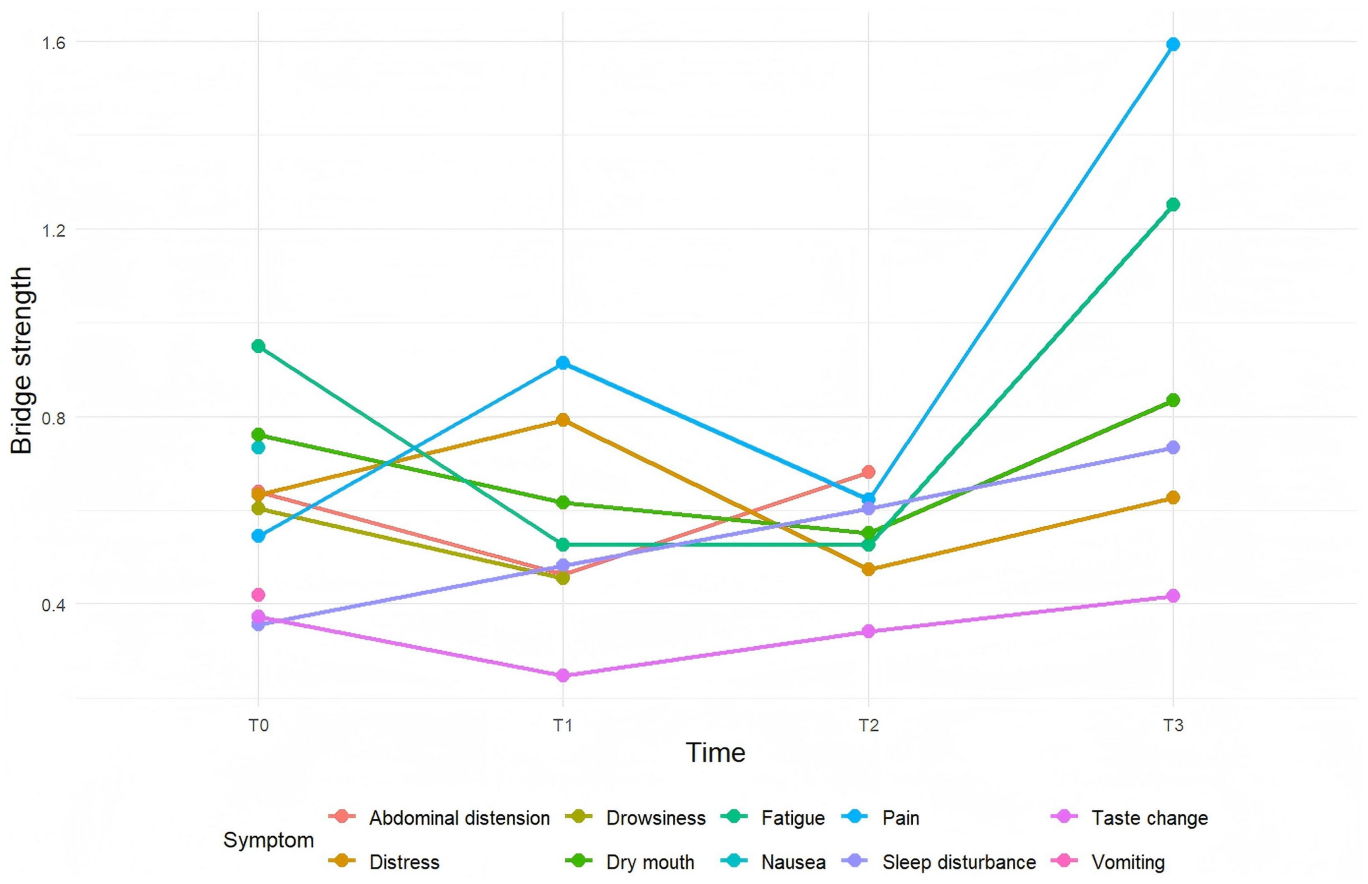
Line chart of bridge symptom metrics in patients with acute pancreatitis. T0: admission day 1; T1: admission day 2; T2: admission day 3; T3: admission day 4.

**Figure 4:**
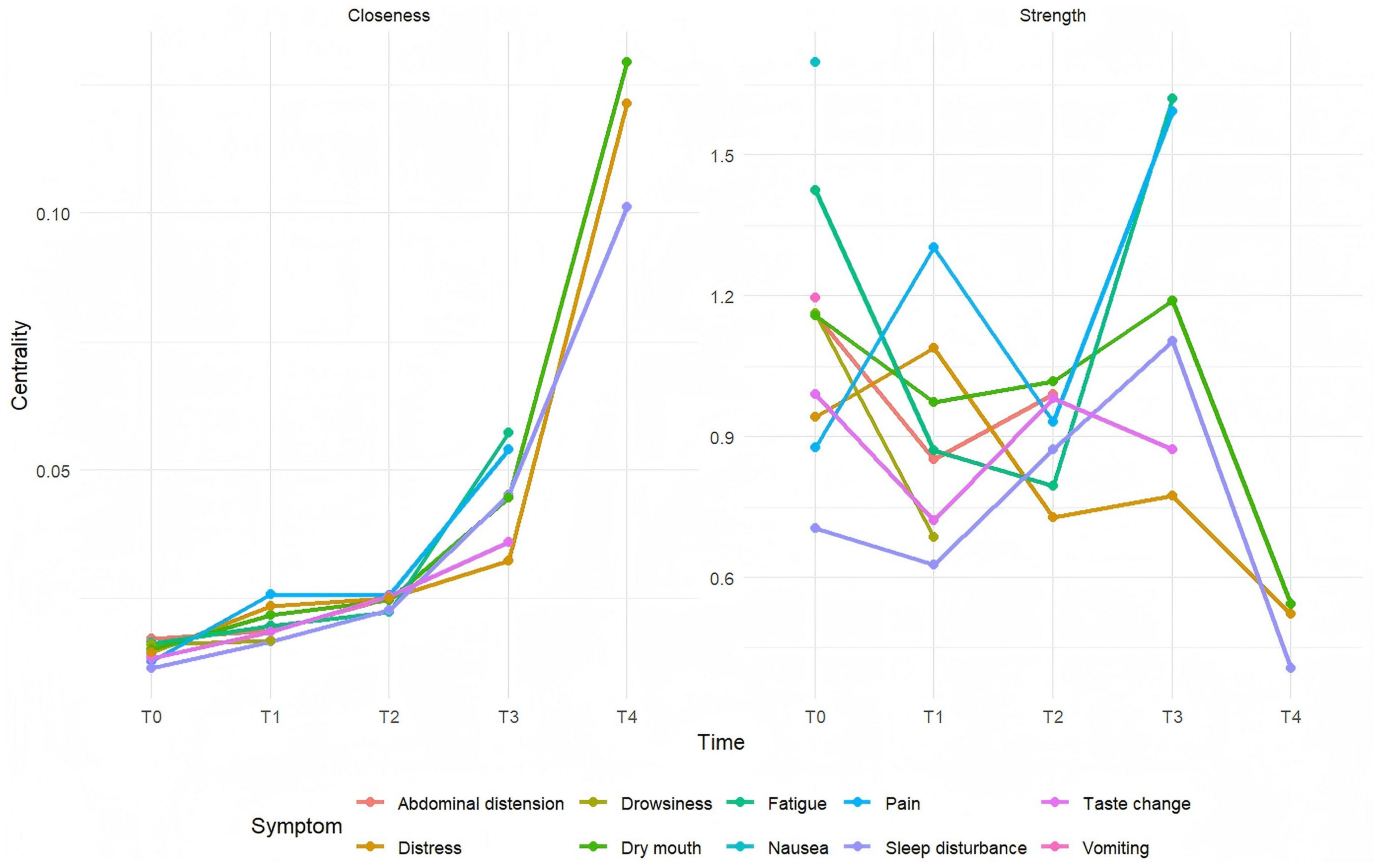
Strength and closeness centrality metrics in symptom networks from T0 to T3. Caption: T0: admission day 1; T1: admission day 2; T2: admission day 3; T3: admission day 4.

The stability coefficients (CS-coefficients) for the T0–T3 symptom networks were 0.284, 0.284, 0.515, and 0.283, respectively. All values exceeded the threshold of 0.25, indicating acceptable stability. Furthermore, the narrow 95% confidence intervals for the edge weights across T0–T3 suggested good accuracy of the estimated symptom networks. Additional details are provided in Figures 2–4.

#### 4.3.2 Temporal trends in symptom networks during hospitalization for acute pancreatitis

Analysis revealed a dynamic evolution in symptom networks over the course of hospitalization. In the initial phase, gastrointestinal symptoms predominated, gradually shifting toward oropharyngeal-psychological discomfort and sleep-fatigue symptom clusters as hospitalization progressed. On hospital day 1 (T0), nausea emerged as the core symptom; however, its incidence decreased rapidly, falling below the 20% threshold by day 2. In contrast, fatigue and abdominal pain consistently demonstrated the highest strength centrality across multiple time points, establishing them as persistent core symptoms. Meanwhile, pain consistently ranked among the symptoms with the strongest bridge strength, identifying it as a key bridge symptom throughout the observation period.

## 5 Discussion

### 5.1 Characteristics of in-hospital symptoms and symptom clusters in patients with acute pancreatitis

During hospitalization, patients with acute pancreatitis (AP) frequently experience multiple co-occurring symptoms, including pain, abdominal distension, nausea, vomiting, dry mouth, taste alteration, fatigue, sleep disturbances, drowsiness, and emotional distress. These symptoms are consistent with the common clinical manifestations of pancreatitis reported in the existing literature (3). The symptoms of patients with acute pancreatitis exhibited dynamic changes over time during hospitalization. The results of this study showed that the incidence rate of pain on the first day of admission was 91.8%, making it the most frequently reported symptom. This finding aligns with both the typical clinical presentation of AP and the incidence reported by Zerem et al. (27). The pain was primarily localized in the mid- and upper-abdominal regions, which is consistent with the underlying pathophysiology of AP (3). Dry mouth was the most prevalent symptom on hospital days 2 and 3 (86 and 64%, respectively). This is likely attributable to patients being maintained nil by mouth, resulting in dryness of the oral mucosa (1). On the fourth day of hospitalization, the incidence of distress peaked at 40.6%, likely due to a combination of factors including persistent pain, discomfort from unresolved gastrointestinal dysfunction, concerns regarding the high risk of disease recurrence, and dietary restrictions (7). This study identified three distinct symptom clusters: a gastrointestinal symptom cluster (pain, abdominal distension, nausea, vomiting), an oropharyngeal-psychological discomfort cluster (dry mouth, altered taste, distress), and a sleep-fatigue cluster (sleep disturbance, drowsiness, fatigue). From a pathophysiological perspective, the emergence of this gastrointestinal symptom cluster is rooted in the inflammatory nature of pancreatitis. The inflammation irritates the gastrointestinal tract, triggering a cascade of dysfunction, intestinal flora dysbiosis, and gas accumulation, which collectively manifest as pain, abdominal distension, nausea, and vomiting (4, 28). Concurrently, the nothing-by-mouth status induces oral symptoms like dry mouth and altered taste. The inability to fulfill basic psychological desires for food and drink thereby generates distress, forming the basis of the oropharyngeal-psychological discomfort cluster (29). Additionally, the protein-energy wasting that is common during the disease course often places patients in a state of negative nitrogen balance, thereby predisposing them to fatigue (30). Moreover, pain and other discomfort can significantly compromise sleep quality, leading to sleep disturbances and drowsiness (31). These factors collectively constitute the sleep-fatigue symptom cluster. The clinical presentation evolves over the course of hospitalization: the gastrointestinal cluster is most prominent initially, later transitioning into the oropharyngeal-psychological and sleep-fatigue clusters. Therefore, we recommend that healthcare professionals perform dynamic symptom assessments and implement targeted interventions accordingly.

### 5.2 Identification of core symptoms in patients with acute pancreatitis during hospitalization

Based on the centrality metrics, the core symptoms at each time point (T0–T3) were identified (Figure 4). Core symptoms exhibit the strongest connections with other symptoms in the network, serving as pivotal hubs (32). They can activate the entire symptom network, triggering a cascade of interrelated symptoms, and act as sentinel indicators for the emergence or exacerbation of other symptoms (33).

Our findings identified nausea as the core symptom with the highest centrality strength on the first day of admission in patients with acute pancreatitis. This aligns with the findings of Yaowmaneerat (34) study, which shows that nausea is a common symptom in pancreatitis. The underlying pathophysiology is multifactorial, potentially encompassing inflammation extending to the gastric posterior wall, intestinal gas accumulation, paralytic ileus, peritonitis, or reflexes triggered by the obstruction of digestive fluid transit (1). Nausea and vomiting constitute primary targets for intervention in the early phase of hospitalization. Current pharmacologic management frequently includes agents such as metoclopramide, haloperidol, and ondansetron. Additionally, non-pharmacological approaches, including acupuncture, acupressure, acupoint injection, and cognitive-behavioral therapy, have also demonstrated beneficial effects (35).

By the second day (T1), pain had become the core symptom. Its pathogenesis is primarily related to inflammatory responses resulting from pancreatic tissue injury. Damaged pancreatic cells release inflammatory mediators that stimulate primary afferent neurons, transmitting signals to the central nervous system and generating pain perception (4). As a nociceptive signal, pain can further trigger the release of neurogenic mediators, creating a vicious cycle that exacerbates pancreatic injury. Therefore, early pain management is crucial. For patients with severe pain, opioid analgesics are preferentially recommended, while a stepwise analgesic strategy may be adopted for those with mild pain (3). Additionally, providing psychological care (e.g., music therapy, verbal reassurance) can help alleviate pain. Clinical healthcare professionals should integrate multidimensional and dynamic pain assessment methods, implement pain intervention plans based on the patient’s pain level and individual tolerance, and promptly adjust the treatment regimen according to therapeutic efficacy and adverse reactions, to achieve rapid and effective pain control.

On the third day (T2), dry mouth emerged as the core symptom, likely due to reduced salivary secretion and mucosal dryness resulting from initial fasting and fluid restriction (29). Studies have shown that when the body cannot meet its physiological needs due to insufficient fluid intake, the resulting thirst not only causes severe physical discomfort in patients but may also further induce negative psychological emotions such as anxiety and irritability (36). Research has shown that the use of oral sprays during fasting can significantly alleviate dry mouth in patients with pancreatitis, improving moisture levels of the oral mucosa and overall patient comfort (37). Therefore, for patients with significant dry mouth, the use of oral moisturizing sprays is recommended during the fasting period, concurrent with a proactive assessment of their clinical status. For those with mild acute pancreatitis, early resumption of oral intake is advised to alleviate these symptoms (38).

On hospital day 4, fatigue emerged as the core symptom. Its pathophysiological basis is likely a hypermetabolic/hypercatabolic state, leading to negative nitrogen balance and disrupted nutrient metabolism. This condition is further exacerbated by insufficient nutrient intake and absorption, which fails to meet the elevated energy demands and consequently intensifies fatigue (30). In clinical practice, nutritional risk screening tools (39) (such as MUST and NRS 2002) should be employed to dynamically assess patients’ nutritional risk. Early oral nutritional support should be initiated as appropriate, with individualized, scientific, and reasonable nutritional support strategies developed by clinical nutrition specialists. Additionally, healthcare professionals should enhance bedside monitoring and health education, guiding patients to properly balance rest and appropriate physical activity while ensuring companionship to prevent adverse events such as falls resulting from fatigue.

In summary, within the symptom management of acute pancreatitis patients, pain and nausea are key targets requiring early and prioritized intervention. It is recommended that clinical staff establish a core symptom-oriented dynamic assessment system and implement personalized interventions following the sequence of “nausea to pain to dry mouth/fatigue,” tailored to each patient’s specific condition. This approach is expected to more effectively alleviate patients’ negative symptom experiences.

### 5.3 Bridge symptoms in hospitalized patients with acute pancreatitis

In this study, bridge symptoms were identified primarily through bridge strength metrics. Bridge symptoms refer to those within a symptom network that connect different symptom clusters or subgroups within the same cluster, possessing a higher capacity for information propagation and enabling mutual influence among distinct symptoms (40). On hospital day 1 (T0), fatigue served as the key bridge symptom, suggesting that interactions between other symptoms and clusters may be mediated through fatigue. This phenomenon may be related to the inflammatory response increasing the body’s energy expenditure and reducing oxygen transport efficiency, thereby triggering fatigue. Furthermore, pain can exacerbate fatigue; the proposed mechanism involves pain stimulating the hypothalamic–pituitary–adrenal (HPA) axis and the sympathetic nervous system, leading to neuroendocrine dysfunction and elevated levels of pro-inflammatory cytokines, which subsequently contribute to fatigue (41). Furthermore, fatigue can predispose patients to psychological discomfort, such as low mood and distress (41). Therefore, fatigue should be prioritized from the first day of hospitalization. We recommend that clinicians implement tailored adaptive nursing interventions for patients with significant fatigue, encompassing dynamic assessment of fatigue severity and contributing factors, followed by personalized measures such as energy management planning and psychological support to mitigate negative inter-symptom reinforcement.

Pain emerged as the primary bridge symptom on days 2 (T1) and 4 (T3) of hospitalization. Pain is frequently associated with sleep disturbances, including poor sleep quality and insomnia (42), and can also precipitate anxiety and irritability (43), As a bridge symptom, pain potentiates interactions between different symptom clusters. A dynamic, stepwise approach to pain assessment is recommended, coupled with a multimodal analgesic strategy integrating pharmacological and non-pharmacological interventions. Treatment plans should be adjusted based on patient response to alleviate pain and mitigate its cross-cluster effects (4).

Abdominal distension was identified as the bridge symptom on day 3 (T2). Research suggests that increasing pancreatic exudates can lead to dysregulation of gastrointestinal hormones, increased intestinal wall permeability, and gut microbiota imbalance. Collectively, these factors often result in abdominal distension (44). Persistent distension can also induce negative emotions like anxiety and irritability, forming a connection with psychological symptom clusters (45). Additionally, severe abdominal discomfort can impair sleep quality. Implementing systematic abdominal assessment and targeted interventions, such as positional adjustments, gastrointestinal decompression when necessary, judicious use of prokinetic agents, and complementary relaxation techniques, is recommended to alleviate distension and associated psychological discomfort.

## 6 Conclusion

Hospitalized patients with acute pancreatitis commonly presented with three relatively stable yet dynamically evolving symptom clusters: a gastrointestinal symptom cluster, a sleep-fatigue cluster, and an oropharyngeal-psychological distress cluster. We found that the patient symptom network dynamically evolved during hospitalization, initially dominated by gastrointestinal symptoms, and gradually shifted to oropharyngeal-psychological discomfort and sleep-fatigue symptom clusters with the prolongation of hospitalization time. According to the calculation results of centrality, fatigue and abdominal pain were found to be persistent core symptoms. Meanwhile, pain was also a key bridging symptom, indicating that these are important intervention targets. Healthcare providers should closely monitor the dynamic evolution of these symptom clusters and their network characteristics. Through early identification of high-risk symptoms and establishment of precise nursing strategies, with particular focus on intervening against core and bridge symptoms, it is possible to effectively alleviate other closely associated symptoms, thereby improving the efficiency of overall symptom management. However, this study was a single-center investigation and thus has certain limitations. Future large-sample, multi-center studies are needed for further validation.

## Data Availability

The original contributions presented in the study are included in the article/supplementary material, further inquiries can be directed to the corresponding author.
